# Quantitative CT texture analysis in predicting PD-L1 expression in locally advanced or metastatic NSCLC patients

**DOI:** 10.1007/s11547-021-01399-9

**Published:** 2021-08-09

**Authors:** Stefano Bracci, Miriam Dolciami, Claudio Trobiani, Antonella Izzo, Angelina Pernazza, Giulia D’Amati, Lucia Manganaro, Paolo Ricci

**Affiliations:** Department of Radiological, Oncological, and Pathological Sciences, Sapienza University of Rome, Viale Regina Elena 324, 00161 Rome, Italy

**Keywords:** Lung cancer, Texture analysis, Chest CT, PD-L1 expression

## Abstract

**Purpose:**

The assessment of Programmed death-ligand 1 (PD-L1) expression has become a game changer in the treatment of patients with advanced non-small cell lung cancer (NSCLC). We aimed to investigate the ability of Radiomics applied to computed tomography (CT) in predicting PD-L1 expression in patients with advanced NSCLC.

**Methods:**

By applying texture analysis, we retrospectively analyzed 72 patients with advanced NSCLC. The datasets were randomly split into a training cohort (2/3) and a validation cohort (1/3). Forty radiomic features were extracted by manually drawing tumor volumes of interest (VOIs) on baseline contrast-enhanced CT. After selecting features on the training cohort, two predictive models were created using binary logistic regression, one for PD-L1 values ≥ 50% and the other for values between 1 and 49%. The two models were analyzed with ROC curves and tested in the validation cohort.

**Results:**

The Radiomic Score (Rad-Score) for PD-L1 values ≥ 50%, which consisted of Skewness and Low Gray-Level Zone Emphasis (GLZLM_LGZE), presented a cut-off value of − 0.745 with an area under the curve (AUC) of 0.811 and 0.789 in the training and validation cohort, respectively. The Rad-Score for PD-L1 values between 1 and 49% consisted of Sphericity, Skewness, Conv_Q3 and Gray Level Non-Uniformity (GLZLM_GLNU), showing a cut-off value of 0.111 with AUC of 0.763 and 0.806 in the two population, respectively.

**Conclusion:**

Rad-Scores obtained from CT texture analysis could be useful for predicting PD-L1 expression and guiding the therapeutic choice in patients with advanced NSCLC.

## Introduction

Lung cancer is the leading cause for cancer death worldwide in both male and female patients with 2,093,876 estimated new cases in 2018 [1]. Eighty-five percent of lung cancer is represented by non-small cell lung cancer (NSCLC) with the majority of patients presenting with advanced disease at diagnosis [2]. Nowadays, the overall survival (OS) of patients with locally advanced or metastatic NSCLC eligible for targeted therapies has significantly increased [3].

Solid cancers, as lung tumor, are able to escape from cytotoxic response through the expression of *Programmed death-ligand 1* (PD-L1) on tumor cell surface that link Programmed cell death protein (PD-1) on lymphocyte surface. As a consequence, tumor-infiltrating lymphocytes (TILs) are inhibited [4]. Immune checkpoint inhibitors are human antibodies that blocks PD-L1/PD-1 linking improving the immune response.

Several clinical studies have shown that Pembrolizumab, a monoclonal antibody that prevents the PD-1/PD-L1 linking, is associated with better disease control and improved OS, with a reduced toxicity profile compared to chemotherapy in patients affected by advanced NSCLC [5–9].

However, the use of Pembrolizumab depends on PD-L1 expression in tumoral cells. According to the National Comprehensive Cancer Network (NCCN) guideline version 4.2021, Pembrolizumab is considered the therapy of choice for patients without mutations of Epidermal Growth Factor Receptor (EGFR) and Anaplastic Lymphoma Kinase (ALK), if PD-L1 is expressed by ≥ 50% of neoplastic cells [3, 5]. Furthermore, patients with PD-L1 expression values between 1 and 49% may still benefit from PD-L1 inhibitor therapy and are addressed to be treated with first-line combined therapy with Pembrolizumab and chemotherapy (carboplatin or cisplatin and pemetrexed) [3, 6, 7].

However, the assessment of PD-L1 expression on a biopsy sample may not reflect the actual biomarker level in the whole tumor. In fact, different studies have shown high variability in the concordance of PD-L1 expression value between biopsy and resection, which according to some authors is around 92%, while for others it is much lower, around 52% [10, 11]. Although this latter study was not clear in reporting the pre-analytic variation, it is clear that this aspect needs further investigation. Consistent with the fact that immunotherapy is currently only indicated for patients with advanced NSCLC, far more biopsies are stained for PD-L1 in daily practice than resection specimens. Thus, PD-L1 testing could be affected by the limited sample size. The fact that approximately 10% of NSCLC tumors respond to PDL1/PD-1 inhibitor despite absent PD-L1 expression may be partly explained by false-negative results on biopsy specimens of tumors heterogeneous for PD-L1 expression. Therefore, a new reliable diagnostic method is currently required in this setting.

Texture analysis (TA) is a technique that provides a quantitative assessment of tumor heterogeneity by analyzing the distribution and correlation of the gray level of single or multiple pixel or voxel in the image analyzed [12, 13]. Several studies have demonstrated the potential role of TA performed on diagnostic images such as computed tomography (CT), magnetic resonance imaging (MRI) and Positron Emission Tomography (PET) in predicting tumors characteristics or response to therapy [14–17].

The aim of our study was therefore to build two predictive models of PD-L1 expression values ≥ 1 and ≥ 50%, respectively, both based on a score formed by radiomic characteristics from baseline contrast-enhanced CT images of patients with advanced NSCLC, in order to noninvasively identify patients who may benefit from immunotherapy as first-line treatment in a pre-operative or pre-biopsy phase.

## Methods

### Patient selection

In this retrospective study, 72 patients with locally advanced or metastatic NSCLC (IIIA–IV stage according to the definition of the American Joint Committee on Cancer TNM staging Manual, 8th Edition) were analyzed at our Institution from April 2018 to September 2019 [18]. The initial cohort included 177 patients, of which 51 were excluded as PD-L1 expression was not available at the time of the study, 35 had neoplastic lesions other than NSCLC and 14 lacked a contrast-enhanced CT scan prior to histological examination. In five patients, it was not possible to analyze DICOM (Digital *Imaging* and COmmunications in Medicine) images for technical reasons. Institutional review board approval was obtained for this study.

### Evaluation of the PD-L1 expression

PD-L1 expression by neoplastic cells was assessed on paraffin sections by immunohistochemistry. Staining was performed with the Ventana PD-L1 SP263 clone (Ventana Medical Systems, Tucson, AZ, USA) using an automatic immunostainer (Benchmark XT, Ventana Medical System, Tucson, AZ, USA).

The PD-L1 expression was evaluated by the tumor proportion score (TPS), which is defined as the percentage of viable tumor cells with at least partial membrane staining relative to all viable tumor cells in the examined section. Positive staining was defined as the presence of membrane staining, strong or weak, complete or incomplete, in a percentage ≥ 1% of neoplastic cells [19]. A minimum of 100 viable tumor cells were evaluated to determine the percentage of stained tumor cells per slide for PD-L1 assessment.

### CT protocol and extraction of radiomic features

CT examinations were performed using 64-row CT (Somatom Sensation Cardiac or Somatom Definition, Siemens, Forchheim, Germany). All tests were performed before and after intravenous administration of a bolus of 1.5 mL/kg of Iomeprol 350 mg/mL (Iomeron 350; Bracco, Milan Italy) at a flow rate of 2.5–4.0 mL/s, followed by an injection of 40 mL saline.

**Fig. 1:**
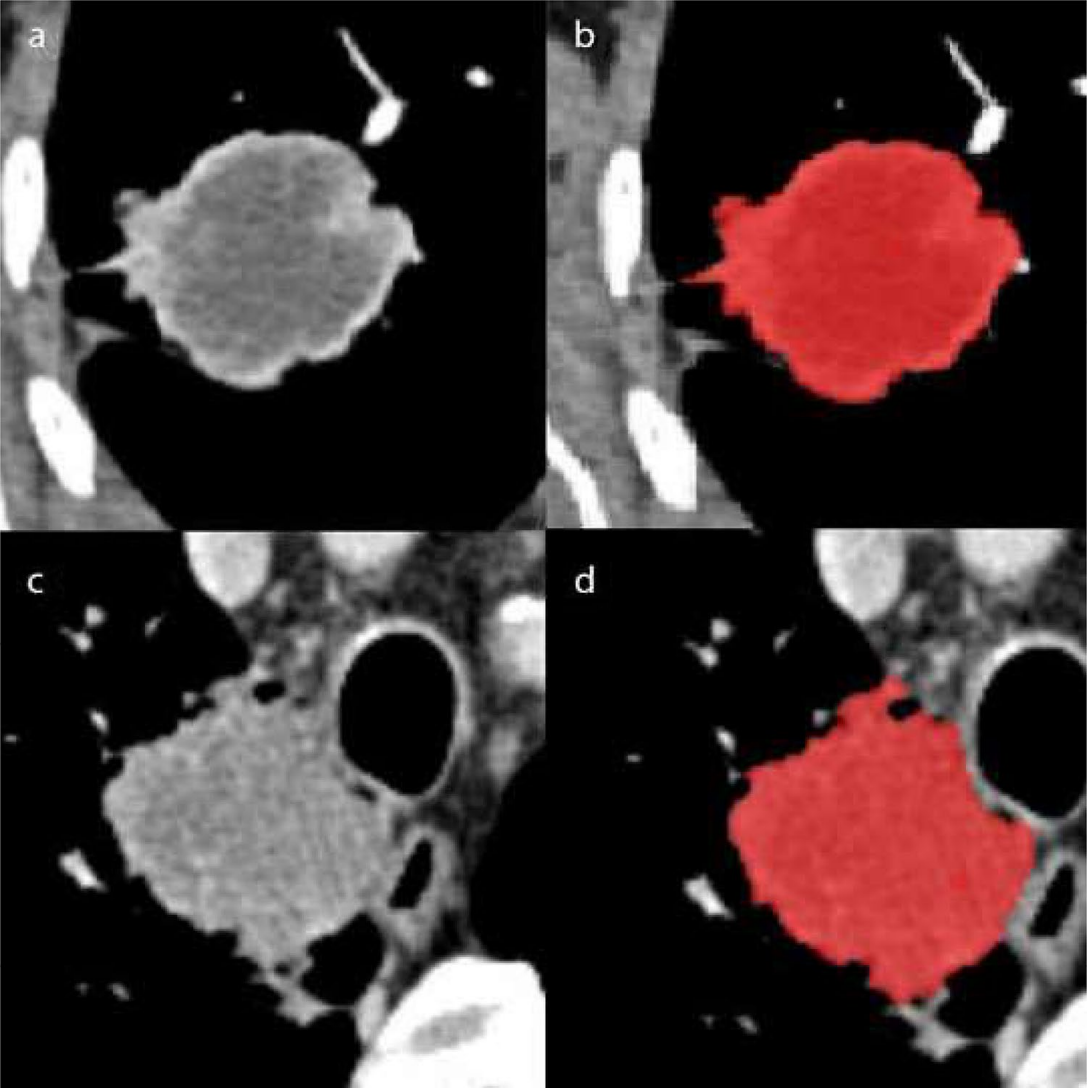
CT axial images showing two primary tumor of the lung, an ADK (**a**) and a SCC (**c**); (**b**) and (**d**) shows the same lesions contoured, respectively

Post-contrast imaging was acquired in the portal phase using an automated bolus-tracking technique with 60-s delay from threshold value (100 HU; with ROI positioned in the descending aorta). The acquisition parameters were set at 120 kV, 200 mAs, pitch 1.5, collimation 0.6 mm, rotation time 0.5 s. All data were reconstructed with a slice thickness of 1.0 mm. The extraction of radiomic features from DICOM files of the portal phase was performed using the LIFEx software (www.lifexsoft.org) [20]. Two radiologists (MD; SB) with 3 and 10 years of experience in chest imaging, respectively, manually and independently contoured the entire volume of the primary lesion on each slice. Each radiologist was blind to the contour selected by other operator. Two contours for each lesion were obtained. Figure 1 shows an example of two different contoured lesions. Then, the software automatically extracted the radiomic features. A total of 48 features were extracted for each contour: 16 first-order features obtained from volumetric and histogram analysis; 7 s-order features using the Gray-Level Co-Occurrence Matrix (GLCM); 25 higher order features, in particular 11 from Gray-Level Length Matrix (GLRLM), 3 from Neighboring Gray-level dependence matrix (NGLDM) and 11 from Gray-Level Zone Length Matrix (GLZLM). We therefore obtained from each lesion a total of 96 features (48 for each contour). The most experienced radiologist (S.B.), several months after the first segmentation session, repeated the segmentation on 20 randomly selected patients, blinded to his previous session.

### Features selection

Before selecting radiomic features for the construction of the two Rad-Score-based predictive models, features obtained from each contour were compared for inter- and intra-observer variability using the Interclass Correlation Coefficient (ICC). To assess intra-observer variability, features obtained at two different times by the same operator (S.B.) on a randomly selected sample of 20 patients were compared by ICC, and all 48 features showed ICC ≥ 0.75. Inter-observer variability was assessed by comparing features extracted from the two contours obtained by the two different operators (S.B. and M.D.) for each lesion in all patients. Features with ICC ≥ 0.75 (40/48 features) were then selected.

The 40 remaining features obtained from the contours performed by the most experienced radiologist (S.B.) were further analyzed to build the Rad-Score. Subsequently, the population was divided into two groups, respectively, consisting of 2/3 (training cohort, formed by 48 patients) and 1/3 (validation cohort, formed by 24 patients) of the patients. Variable selection and the construction of the two predictive models (for PDL1 values < or ≥ 1% and < or ≥ 50%) was carried out on the training cohort and was subsequently tested on the validation cohort. Due to the high collinearity of features, the Least Absolute Shrinkage and Selection Operator (LASSO) regression method was performed on the 40 features from the training cohort to select variables. This method introduces a penalty called tuning parameter (*λ*) capable of penalizing the estimated coefficient (*β*) of the variables in the model so that the lower the value of *λ*, the more variables will be selected [21, 22]. The “glmnet” package from the R software was used to perform the LASSO regression.

### Statistical analysis

Mann–Whitney's *U* test was used to compare continuous variables, while categorical variables were compared with Fisher's *F* test or Chi-squared test (*χ*^2^). Binary logistic regression was used after LASSO regression to further select variables with *p *value < 0.10 which therefore formed the Radiomics Score (Rad-Score) that was computed for each patient through a linear combination of selected features weighted by their respective coefficients [22–24]. A further binary logistic regression was used to test the Rad-Score with patient and tumor variables such as age, gender, smoking status and type of histology. The model created was analyzed using the receiver operating characteristic (ROC) curve, and the best cut-off was calculated with the Youden index. The Area under the ROC Curve (AUC), sensitivity, specificity, positive predictive value (PPV) and negative predictive value (NPV) were then calculated for the best cut-off. The model was then tested on the validation cohort. All the steps described were performed two times, i.e., for PD-L1 values < or ≥ 1% and for values < or ≥ 50%. Statistical analyses were performed using the SPSS v.25 software for Macintosh (IBM, Armonk, NY, USA).

## Results

### Patient and tumor characteristics

*Immunohistochemistry (IHC)* was performed on biopsy samples in 28 patients (38.9%), on endobronchial ultrasound (EBUS)-guided needle aspirates in 19 patients (26.4%) and on surgical specimens (35 cases) from lobectomy in 11 patients (15.3%), wedge resection in 12 patients (16.6%) and pneumonectomy in 2 patients (2.8%), respectively. Overall, patients mean age was 66.8 years, of whom 35 (48.6%) were male and only 12 (16.7%) had never smoked. Primary tumor lesions had a mean maximum diameter of 45.6 ± 22.3 mm. They were located in the upper lobes in 61.1% of cases, and in the right lung in 70.8% of patients. The most frequent histotype was adenocarcinoma (ADK) (47 patients, 65.3%), while squamous-cell carcinoma (SCC) had been diagnosed in 25 patients (34.7%). At diagnosis, 22 patients (30.6%) were in stage IIIA, 20 patients (27.8%) in stage IIIB, 6 patients (8.3%) in IIIC and 24 patients (33.3%) in stage IV.

Twenty-three patients had a PD-L1 expression < 1% and 48 patients ≥ 50%. Patient and tumor characteristics were overall well balanced among the groups (Table 1).

**Table 1:** Baseline population characteristics

Characteristics	Total (*n* = 72)	PDL-1 < 1% (*n* = 23)	PDL-1 $\ge$ 1% (*n* = 49)	*p *value	PDL-1 < 50% (*n* = 48)	PDL-1 $\ge 50$% (*n* = 24)	*p *value
*Age*	68.8 ± 9.7	64.4 ± 11.1	67.9 ± 8.9	0.174	66.1 ± 10.2	68.2 ± 8.7	0.318
*Sex*				0.927			0.095
M	35 (48.6%)	11(47.8%)	24 (49.0%)		20 (41.7%)	15 (62.5%)	
F	37 (51.4%)	12 (52.2%)	25 (51.0%)		28 (58.3%)	9 (37.5%)	
Smoking status				0.429			0.180
Yes	60 (83.3%)	18 (78.3%)	42 (85.7%)		38 (79.2%)	22 (91.7%)	
No	12 (16.7%)	5 (21.7%)	7 (14.3%)		10 (20.8%)	1 (8.3%)	
*T Diameter* (mm)	45.6 ± 22.3	46.3 ± 27.4	45.4 ± 19.8	0.937	42.8 ± 22.6	51.4 ± 20.9	0.150
*Location*				0.113			0.494
Superior	44 (61.1%)	11 (47.8%)	33 (67.3%)		28 (58.3%)	16 (66.7%)	
Inferior	28 (38.9%)	12 (52.2%)	16 (32.7%)		20 (41.7%)	8 (33.3%)	
*T side*				0.473			0.582
Right	51 (70.8%)	15 (65.2%)	36 (73.5%)		33 (68.8%)	18 (75.0%)	
Left	21 (29.2%)	8 (34.8%)	13 (26.5%)		15 (31.3%)	6 (25.0%)	
*Histology*				0.601			0.381
ADK	47 (65.3%)	16 (69.6%)	31 (63.3%)		33 (68.8%)	14 (58.3%)	
SCC	25 (34.7%)	7 (30.4%)	18 (36.7%)		15 (31.3%)	10 (41.7%)	
*Clinical stage*				0.769			0.706
IIIA	22 (30.6%)	7 (30.4%)	15 (30.6%)		15 (31.3%)	7 (29.2%)	
IIIB	20 (27.8%)	8 (34.8%)	12 (25.5%)		15 (31.3%)	5 (20.8%)	
IIIC	6 (8.3%)	2 (8.7%)	4 (8.2%)		4 (8.3%)	2 (8.3%)	
IV	24 (33.3%)	6 (26.1%)	18 (36.7%)		14 (29.2%)	10 (41.7%)	

### *Rad-Score for PD-L1 expression level* ≥ *50% and validation of the model*

LASSO regression method was able to shrink variables from 40 to 4, namely Skewness, Sphericity, GLZLM_LGZE and GLZLM_GLNU. In the training cohort, patients with PD-L1 ≥ 50% had significantly lower Skewness (1.98 vs. − 2.97, < 50% vs. ≥ 50%, *p* value: < 0.01), while the other variables had no statistically significant differences (Sphericity: 0.935 vs. 0.922, < 50% vs. ≥ 50%, *p* value: 0.27; GLZLM_LGZE: 0.200 vs. 0.134, < 50% vs. ≥ 50%, *p* value: 0.119; GLZLM_GLNU 595.41 vs. 367.42, < 50% vs. ≥ 50%, *p* value: 0.503). Binary logistic regression further selected Skewness and GLZLM_LGZE, as shown in Table 2. The Rad-Score was then obtained by applying the following formula (extrapolated from the binary logistic regression analysis) to each patient of the training cohort:

$${\text{Rad - Score}}( \ge {5}0\% ): \, - {1}.{192} + \left( { - 0.{937} \times {\text{Skewness}}} \right) + \left( { - {11,259,862} \times {\text{GLZLM}}\,{\text{LGZE}}} \right)$$

**Fig. 2:**
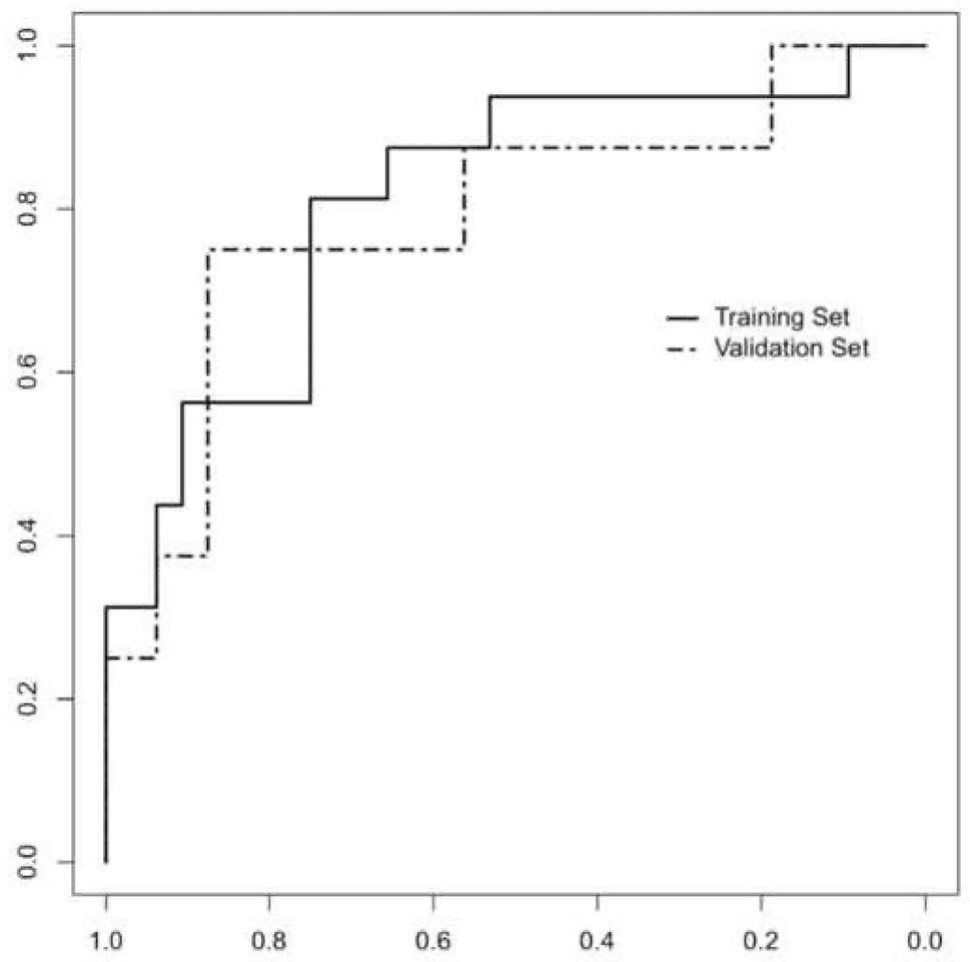
Classifiers’ performance on predicting PD-L1 expression level ≥ 50% in training set and validation set

Subsequently, the Rad-Score was included in a model along with patient and tumor variables, in which we observed that the Rad-Score was the only significant variable between the two populations (PD-L1 < 50% vs. ≥ 50%) (Table 3). Then, the ROC curve was obtained for the model that was formed by the Rad-Score alone presenting an AUC of 0.811 (95% CI 0.676–0.945). The optimal cut-off value calculated was − 0.745 with a sensitivity of 83%, specificity of 75%, PPV of 61.9% and NPV of 88.9%. The Rad-Score was then applied to the validation cohort obtaining an AUC of 0.789 (95% CI 0.579–0.999) (Fig. 2). Overall, the Rad-Score was significantly lower in patients with PD-L1 < 50% vs. ≥ 50% in the training cohort (− 1.591 vs. 0.0846; *p* value < 0.001), while in the validation cohort showed tendency to significance (− 2.75 vs. 0.26; *p* value: 0.06).

**Table 2:** Features selected after LASSO regression for PD-L1 expression values ≥ 50%

Variable	OR	95% CI	*p *value
Skewness	0.248	0.090–0.683	< 0.010
Sphericity	0.001	0.001–0.813	0.162
GLZLM_LGZE	0.012	0.090–0.906	0.049
GLZLM_GLNU	0.998	0.994–1.001	0.164

**Table 3:** Predictive model for PD-L1 expression values ≥ 50%

Variable	$\beta$	OR	95% CI	*p *value
Rad-Score	1.313	3.718	1.29–10.65	0.014
Age	− 0.019	0.981	0.90–1.07	0.658
Sex (M vs. F)	0.331	1.392	0.20–9.51	0.736
Smoking status (Yes vs. No)	− 1.382	0.251	0.02–3.80	0.319
Histology ADC versus SCC	− 0.093	0.911	0.16–5.02	0.915
T diameter	− 0.021	0.980	0.93–1.03	0.420

### *Rad-Score for PD-L1 expression level* ≥ *1% and validation of the model*

LASSO regression reduced the number of variables from 40 to 6; in particular, Skewness, Sphericity, Conv_Q3, GLZLM_GLNU, NGLDM_Coarsness and GLRLM_LRLGE were selected. In the training cohort, there were no significant differences in the value of the selected variables between the two groups (Skewness − 1.95 vs. -− .51, < 1% vs. ≥ 1%, *p* value: 0.886; Sphericity: 0.944 vs. 0.923, < 1% vs. ≥ 1%, *p* value: 0.08; Conv_Q3 82.1 vs. 80.2, < 1% vs. ≥ 1%, *p* value: 0.843; GLZLM_GLNU 850.24 vs. 337.99 < 1% vs. ≥ 1%, *p* value: 0.123; NGLDM_Coarsness 0.0014 vs. 0.0006 < 1% vs. ≥ 1%, *p* value: 0.120; GLRLM_LRLGE 0.00037 vs. 0.00018 < 1% vs. ≥ 1%, *p* value: 0.210). Binary logistic regression selected Sphericity, Conv_Q3, GLZLM_GLNU and Skewness (Table 4). The Rad-Score was then built by applying the following formula extrapolated from the binary logistic regression:

$$\begin{aligned} {\text{Rad - Score }}( \ge \,{1}\% ) & :{17,}0{63}\, + \,\left( { - 0.{72}\, \times \,{\text{Skewness}}} \right)\, + \,\left( { - {16,777}\, \times \,{\text{Sphericity}}} \right)\, \\ & \quad + \,\left( { - 0.0{22}\, \times \,{\text{Conv}}\_{\text{Q3}}} \right)\, + \,( - 0.00{1}\, \times \,{\text{GLZLM}}\_{\text{GLNU}}). \\ \end{aligned}$$

**Fig. 3:**
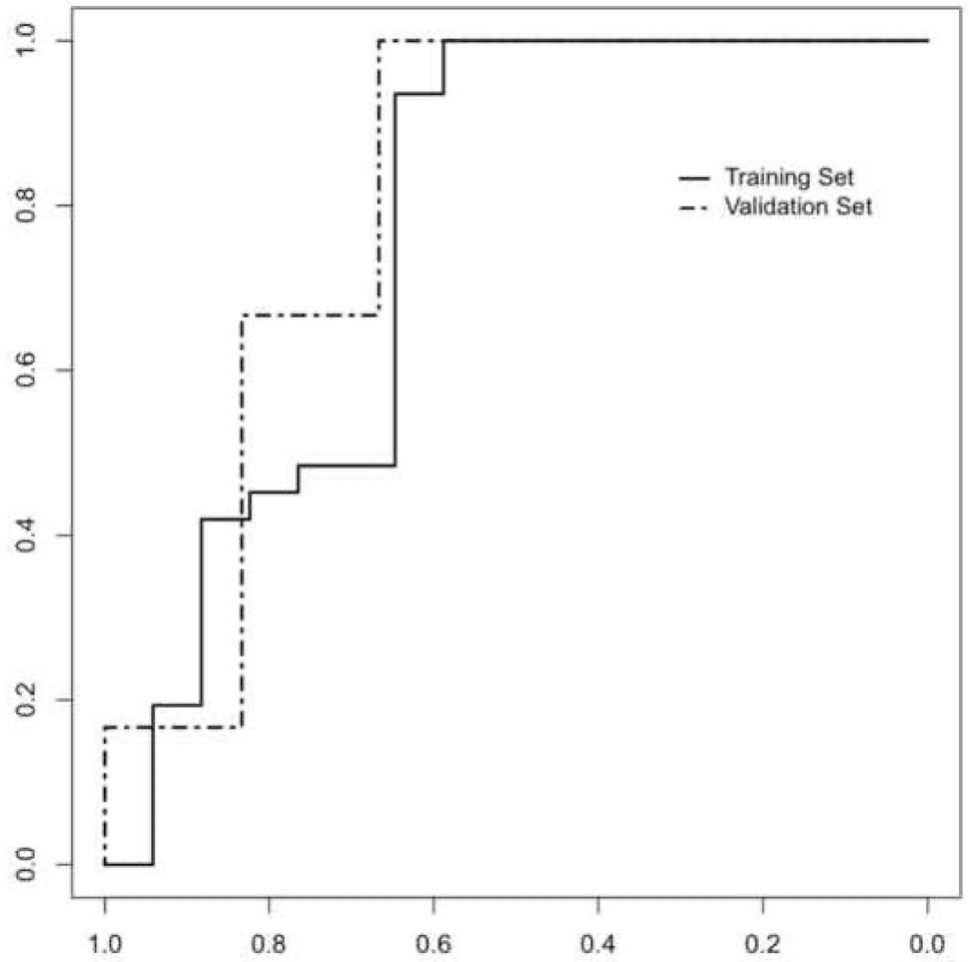
Classifiers’ performance on predicting PD-L1 expression level ≥ 1% in training set and validation set

Even in this situation, the Rad-Score was the only significant variable of the model (Table 5). The ROC curve obtained from the model had an AUC of 0.763 (95% CI 0.597–0.928). The optimal cut-off value was 0.111 and had a sensitivity of 100%, specificity of 58.8%, PPV of 81.6% and NPV of 100%. The Rad-Score was then applied to the validation cohort that had an AUC of 0.806 (0.548–1.000) (Fig. 3). Overall, the Rad-Score was significantly lower in patients with PD-L1 < 1% vs. ≥ 1% both in the training cohort (− 0.026 vs. 1.263; *p* value: 0.002) and in the validation cohort (− 0.41 vs. 1.50; *p* value 0.03).

**Table 4:** Features selected after LASSO regression for PD-L1 expression values ≥ 1%

Variable	OR	95%CI	*p* value
Skewness	0.400	0.143–1.123	0.032
Sphericity	0.003	0.001–0.641	0.047
Conv_Q3	0.966	0.935–0.998	0.037
GLZLM_GLNU	0.997	0.995–1.000	0.037
NGLDM_Coarsness	0.002	0.001–1.821	0.111
GLRLM_LRLGE	0.003	0.001–56,483	0.413

**Table 5:** Predictive model for PD-L1 expression values ≥ 1%

Variable	$\beta$	OR	95% CI	*p *value
Rad-Score	1.171	3.226	1.24–8.43	0.017
Age	0.050	1.051	0.98–1.13	0.182
Sex (M vs. F)	0.483	1.621	0.29–8.79	0.576
Smoking status (Yes vs. No)	− 0.065	0.937	0.13–7.01	0.949
Histology ADC versus SCC	− 0.824	2.279	0.40–12.94	0.353
T diameter	0.001	0.962	0.96–1.04	0.962

## Discussion

Evaluation of PD-L1 expression has become of crucial importance in patients with locally advanced or metastatic lung cancer because of the development of inhibitors of the PD-1/PD-L1 system [2–7]. The KEYNOTE-024 study showed that Pembrolizumab is associated with longer Progression-free survival (PFS) (median 10.3 vs. 6.0 months; HR 0.50, 95% CI 0.37–0.68, *p* value < 0.001) and OS (6 months OS of 80.2% vs. 72.4%, HR 0.60, 95% CI 0.41–0.89, *p *value = 0.005) with a better tolerability profile compared to CHT in patients affected by advanced NSCLC and expression levels of PD-L1 ≥ 50% [5]. In fact, according to NCCN guidelines, Pembrolizumab should be used as first-line monotherapy in these patients. [3] Furthermore, on the basis of the KEYNOTE-042 and KEYNOTE-021 studies, the NCCN guidelines have recently been modified allowing PD-L1 inhibitor to be prescribed even in patients with PD-L1 expression between 1 and 49%, indicating first-line combination therapy with Pembrolizumab and chemotherapy [3, 6, 7].

Several studies investigated the use of radiomic models in predicting EGFR status in patients with lung cancer [14, 16, 25, 26]. Ozkan et al., demonstrated that radiomic features could be useful in differentiating EGFR mutation type in pulmonary ADK patients [25]. Another study by Liu et al. [26] showed that texture features could differentiate patients with EGFR mutations from wild-type patients and that radiomic model performance can be increased by adding clinical variables.

In the present study, in order to identify patients with different level of PD-L1 expression requiring different first-line therapies, we constructed two radiomic-based predictive model of PD-L1 expression for values ≥ 1% and ≥ 50%, respectively. In particular, Skewness was selected for both models, while GLZLM_LGZE became part of PD-L1 ≥ 50% model and Sphericity, Conv_Q3 and GLZLM_GLNU were included in PD-L1 ≥ 1% model. Jiang et al. recently published a paper in which they obtained CT- and PET-derived radiomic model for PD-L1 expression of both ≥ 1% and ≥ 50% [27]. Their CT model was formed by several radiomic features including GLZLM_GLNU and interquartile range, while Skewness entered only in the PET-based model.

In the present paper, we found that tumors with expression of PD-L1 ≥ 1% and ≥ 50% have lower Skewness than those with expression of PD-L1 < 1% or < 50%; a negative Skewness is characterized by the peak of pixel distribution shifted on the right with the tail on the left side in the histogram, meaning that the majority of pixels have high value. Moreover, patients with PD-L1 ≥ 50% have also lower GLZLM_LGZE that represents the distribution of low gray-level zones in three-dimensional (3D) space. Differently, tumors with PD-L1 ≥ 1% showed less Skewness, less Sphericity and less GLZLM_GLNU, which represents non-uniformity of the gray-level zones. This could mean that these lesions are less spherical and have a more uniform microstructure than tumors with PD-L1 < 1%.

There are only few papers that have applied radiomics to predict PD-L1 expression. Jiang et al. conducted a study on 399 NSCLC patients and built a radiomic model based on CT, PET and PET-CT images capable of predicting the expression of PD-L1 [27]. The author concluded that the model obtained with CT features had a better diagnostic performance than the one obtained with PET features. In order to compare the radiomic data with the true expression of PD-L1, the authors decided to include only patients undergoing surgery in that study, so that 75% of patients were in stage I–II and only 25% of patients had locally advanced (86 patients) or metastatic (12 patients) disease. However, it should be considered that inhibitors of the PD-1/PD-L1 system are used only in advanced NSCLC and therefore, the expression of PD-L1 analyzed may not reflect the actual condition of the target population.

Several studies with multiple PD-L1 clones have reported intratumoral and intertumoral heterogeneity of PD-L1 expression that could result in discrepant results between different specimen types (i.e., resection vs. biopsy and the primary tumor vs. metastasis) [11, 19, 28]. In addition, PD-L1 expression can be affected by chemotherapy and/or radiation therapy [29].

Conversely, in our study we analyzed only patients with locally advanced or metastatic NSCLC, a choice that reflects the real population to which immunotherapy is addressed, even if in the most cases these patients were not submitted to surgery and therefore, PD-L1 expression obtained on biopsy sample could not reflect its real expression.

A study by Yoon et al. performed on 153 patients affected by advanced lung ADK demonstrated that quantitative CT radiomic features could be useful in predicting PD-L1 expression compared to qualitative CT characteristics alone; in particular, a prediction model composed of both clinical and radiomic features could facilitate the assessment of PD-L1 expression [24]. In that study, the authors analyzed only one PD-L1 cut-off value as patients were considered as PD-L1 positive for value of expression ≥ 50%. Differently, we constructed two different models for the two different cut-off values of PD-L1 currently used in the clinical application of the PD-1/PD-L1 inhibitors, namely 50% and 1%. Moreover, as the authors stated, in that study the model found was not validated on an independent cohort making the results less generalizable. Finally, none of the radiomic features presents in the Rad-Score matched with the radiomic features we included in our models. These differences are probably due to several reasons, such as having analyzed different groups of radiomic variables (e.g., GLZLM characteristics not analyzed in the study by Yoon et al.) and different histotypes of lung cancer (we also included patients with SCC and not only patients with ADK).

A recent study by Sun et al., conducted on 390 patients, showed that PD-L1 positivity (TPS ≥ 50%) could be predicted using a model consisting of both the Rad-Score and clinicopathological characteristics [30]. Otherwise, in our study we decided to analyze the models formed by the Rad-Score alone since this was the only statistically significant variable among the patient groups. Moreover, also in this study both patients with localized and advanced disease were analyzed (in the training cohort, almost 40% of patients were in stage I and II). Finally, feature extraction was performed only on unenhanced CT images, leading to potential omission of significant tumor features. In fact, even if analysis on non-contrast CT allows a better assessment of the raw structure, contrast medium administration in our opinion may better highlight differences in tumor heterogeneity. Intratumoral inhomogeneity after contrast medium depends on neoangiogenesis and necrosis, markers of tumor biological behavior, which in turn affect heterogeneity in pixel density and consequently radiomic features.

The present study has, however, some limitations. First of all, the small number of patients included has probably affected the results; in fact, for the cut-off value of 50% we found that the Rad-Score showed only a tendency to significance in the validation cohort (< 50%: − 2.75 vs. ≥ 50%: 0.26; *p* value 0.06).

Additionally, most patients underwent biopsy rather than surgery. In this way, the PD-L1 value obtained probably did not fully reflect the true PD-L1 expression of the entire lesion, although it must be considered that patients with locally advanced or metastatic disease are rarely treated with surgery. Furthermore, we have not yet analyzed the clinical data of response to therapy and the possible role of the radiomic model to predict this outcome, while several studies have recently shown that texture analysis is able to predict the response to immunotherapy [17].

## Conclusions

The use of a Rad-Score is helpful for accurately predicting the expression status of PD-L1 for both the cut-off value of 50% and 1%, allowing identification of two categories of patients requiring different therapeutic strategies. This could become an indispensable tool in guiding therapeutic choice when the real expression of PD-L1 is not known either because patients are not suitable for the invasive procedure or because the tissue sample obtained is not adequate for IHC or because PD-L1 expression may change over time and not be biologically relevant at the time of sampling. In this setting, CT examination with texture analysis has the advantage both of being a noninvasive examination that analyzes the entire tumor volume and that it can be repeated at different times to assess possible changes in gene expression.

## Data Availability

The datasets analyzed during this study are available from the corresponding author upon reasonable request.

## Code availability

Quantitative CT texture analysis was performed by using a dedicated software (LIFEx 6.00, www.lifexsoft.org).
